# Pilot data of right ventricular myocardial T1 quantification by free-breathing fat-water separated dark blood saturation-recovery imaging

**DOI:** 10.1186/1532-429X-17-S1-Q23

**Published:** 2015-02-03

**Authors:** Ee Ling Heng, Peter Kellman, Michael A Gatzoulis, James Moon, Peter Gatehouse, Sonya V Babu-Narayan

**Affiliations:** 1 NIHR Cardiovascular Biomedical Research Unit, Royal Brompton Hospital, London, UK; 2 National Heart & Lung Institute, Imperial College London, London, UK; 3 National Heart, Lung, and Blood Institute, National Institutes of Health, Bethesda, MD, USA; 4 Department of Adult Congenital Heart Disease, Royal Brompton Hospital, London, UK; 5 Cardiovascular Magnetic Resonance Unit, Royal Brompton Hospital, London, UK; 6 Heart Hospital Imaging Centre, The Heart Hospital, UCLH & UCL, London, UK

## Background

Right ventricular (RV) T1 quantification is desirable in managing congenital heart disease and pulmonary hypertension patients where RV fibrosis is implicated. RV T1 quantification is technically difficult because of the thin trabeculated mobile wall of complex geometry, impacted by adjacent blood and epicardial fat, plus proximity to sternal wires in some cases. Prior work has measured RV T1 by multi-shot segmented imaging^1^, further by IDEAL fat-water separation in SASHA extended to suppress blood signal by inflow of saturated blood.^2^ We present initial results by single-shot imaging with motion-corrected (MoCo) averaging aiming to: 1) reduce ghost artifacts arising in a segmented scan, 2) apply fat-water separation, 3) null blood within the RV, 4) facilitate anchor image acquisition and 5) permit free-breathing acquisition.

## Methods

Data for five healthy volunteers was acquired during free-breathing (FB) on a 1.5T Siemens Avanto by MoCo averaging of fat-water separated single-shot scans.^3^ Dark blood motion-sensitized^4^ preparation (MSPrep) parameters and feasibility were investigated.

**Figure 1:**
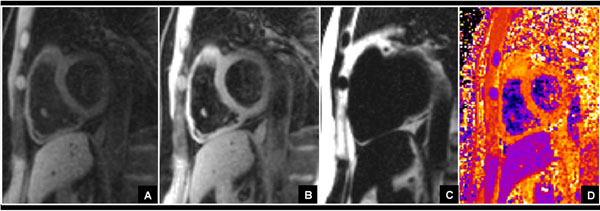
Normal volunteer RV T1 by fat-water separated, MSPrep dark blood imaging: A) MoCo averaged water-only image at Ts 600ms, B) MoCo averaged water-only anchor image at same window/level, C) MoCo averaged fat only image, D) T1 map generated from registration and 2-point fit of the six images per sampling scheme.

Saturation recovery sampling^5^ was applied to FB acquisitions of a single short-axis slice across the RV free wall. The sampling comprised four 20-cycle scans at Ts≈600ms and two anchor scans at Ts>6sec (20 cycles acquired with long recovery gaps), all repeated three times per subject for intra-session reproducibility (Figure 1). Complex MoCo averaging^6^ was configured at fixed 50% acceptance i.e. the 10 shots at most similar respiratory phase. Imaging parameters were: TE=1.0, 2.7, 4.3ms, FA 20°, FOV 360x270mm, 6x1.9x2.5mm acquired voxels, TGRAPPA rate 3, requiring a diastolic shot duration 190ms.

Optimised BIR-4 saturation efficiency η was <0.5% of M_0_ over typical 1.5T cardiac B_0_ and B_1_ distortion. The MSPrep aimed to null blood signal by through-slice velocity-sensitized dephasing without losing myocardial signal, by adjustable time of application and velocity-sensitivity Field-of-Speed^4^. Mean RV free wall and septal T1s were independently measured by two observers from 2-parameter fit pixelwise maps assuming η=1.

## Results

Moderate free-breathing reproducibility of RV T1 was demonstrated (Table 1). There was usually an underestimate of myocardial T1^7^ and often a currently unexplained non-uniformity of T1 across the heart. The MSPrep generally nulled blood when applied typically 50ms after the start of diastasis, with FoS 10-25cm/s. However, subject-specific optimisation of both MSPrep parameters was necessary to null blood while avoiding myocardial signal loss.

**Table 1:** Myocardial T1 values in volunteers reported as mean ± standard deviation (SD) with coefficient of variation (CoV = SD/mean x100%). FB: free breathing, RV: right ventricle, LV: left ventricle

	Observer 1			Observer 2		
	Mean T1 (ms)	SD	CoV (%)	Mean T1 (ms)	SD	CoV (%)

FB RV free wall	1130	62	5.5	1139	61	5.4

FB LV septum	1027	57	5.6	1025	62	6.0

## Conclusions

Although FB RV T1 quantification is feasible with the proposed method, further technical development work is required and underway towards improved precision and accuracy.

## Funding

British Heart Foundation, NIHR Cardiovascular Biomedical Research Unit of Royal Brompton & Harefield NHS Foundation Trust and Imperial College London.
